# Guidelines for the diagnosis and management of carcinoid tumours. Part 1: The gastrointestinal tract. A statement from a Canadian National Carcinoid Expert Group

**DOI:** 10.3390/curroncol13020006

**Published:** 2006-04

**Authors:** J. Maroun, W. Kocha, L. Kvols, G. Bjarnason, E. Chen, C. Germond, S. Hanna, P. Poitras, D. Rayson, R. Reid, J. Rivera, A. Roy, A. Shah, L. Sideris, L. Siu, R. Wong

**Affiliations:** * Ottawa Hospital Regional Cancer Centre, Ottawa, Ontario; † London Regional Cancer Centre, London, Ontario; ‡ H. Lee Moffitt Cancer Centre, Tampa, Florida, U.S.A; § Toronto–Sunnybrook Regional Cancer, Toronto, Ontario; || University Health Network, Princess Margaret Hospital, Toronto, Ontario; # Sudbury Regional Hospital–Laurentian, Sudbury, Ontario; ** Sunnybrook and Women’s College Health Sciences Centre, Toronto, Ontario; †† CHUM–Hospital St-Luc, Montreal, Quebec; ‡‡ Queen Elizabeth II Health Sciences Centre, Halifax, Nova Scotia; §§ London Health Sciences Centre–Victoria Campus, London, Ontario; |||| MUHC–Royal Victoria Hospital, Montreal, Quebec; ## B.C. Cancer Agency–Vancouver Cancer Centre, Vancouver, British Columbia; *** Hôpital Maisonneuve–Rosemont, Montreal, Quebec; ††† St. Boniface General Hospital, Winnipeg, Manitoba

**Keywords:** Carcinoid tumour, carcinoid syndrome, guidelines, clinical management

## Abstract

Carcinoid tumours are relatively rare and, in general, slow growing. They can be “non-functioning” tumours, presenting as a tumour mass, or “functioning” tumours secondary to the production of several biopeptides leading to the carcinoid syndrome. Though these tumours represent 0.25% of an oncology practice, a proper understanding of the clinical course of the disease and of the importance of appropriate diagnostic and therapeutic measures is very important. Proper patient management can lead to cure, particularly if the tumour can be fully resected, or to long-term palliation with medical treatment or cytoreductive surgery, or both, with significant prolongation of survival. A good understanding of the use of somatostatin analogues to achieve effective symptomatic control and of the importance of adequate follow-up and cardiac monitoring to prevent or effectively treat cardiac complications can contribute significantly to optimal control of this complex disease, ultimately improving the quality of life of affected patients. This article, developed by a group of Canadian experts, provides a framework that will assist clinicians in taking an optimal approach to managing their patients with carcinoid tumour.

## 1. INTRODUCTION

Carcinoid tumours are relatively rare and slow-growing tumours that originally were thought to be benign. However, they may become aggressive and resistant to therapy 1. The tumours can secrete several biologically active substances, including serotonin (5-hydroxytryptamine), kallikrein, histamine, prostaglandins, adrenocorticotropic hormone, gastrin, calcitonin, and growth hormone, among others. The gastrointestinal tract accounts for about two thirds of carcinoids. Within the gastrointestinal tract, most tumours occur in the small intestine (41.8%), rectum (27.4%), and stomach (8.7%) 2. A delay of several years frequently occurs before a diagnosis of carcinoid tumour is made.

In Canada, carcinoid tumours represent less than 0.25% of the oncology patient load. Practice varies widely across centres, but a few specialized centres with large loads are emerging (Novartis Pharmaceuticals Canada. Canadian Carcinoid Survey 2005. Unpublished data). In view of the rare occurrence of carcinoids, the experience of physicians and surgeons with the tumour is limited, suggesting a need for information that can facilitate diagnosis and management.

Recently, European guidelines were developed by the Nordic NE Tumour Group and the European Neuroendocrine Tumour Society 3–5. The National Comprehensive Cancer Network guidelines 6 were published in the United States, and in the United Kingdom, guidelines were published on behalf of the UKNETwork for Neuroendocrine Tumours 7. However, in Canada, no consensus or guideline for diagnosing and managing this condition has been established. The Canadian National Carcinoid Expert Group, whose members have considerable experience in the field, was convened to study the practices pertaining to carcinoid tumours in Canada and to develop guidelines to facilitate diagnosis and treatment. The group members reached a consensus (see Table I for consensus categories), and the present guidelines agree in general with those from Europe and the United States.

## 2. EPIDEMIOLOGY

Carcinoid tumours have a low incidence rate of 1.9 per 100,000 8. The overall incidence appears to have increased since the early 1970s, which may at least partly reflect better diagnosis and awareness. However, in Ontario, the incidence rose from 2 per million in 1964 to 22 per million in 2002 (W. Kocha. Personal communication, June 2005), and the speculation is that the advent and increasing use of proton pump inhibitors is a major contributory factor to that increased incidence 9.

Distant metastases may be evident at the time of diagnosis in 12.9% of patients, but better diagnostic techniques have contributed to improved survival rates 2,8. Evolution in diagnostic methods—and in medical and surgical therapies—has led in recent years to more active care and a more favourable prognosis for patients 10. A five-decade analysis of 13,715 carcinoid tumours showed an overall 5-year survival rate of 67.2%, with the best survival rates being recorded for patients with rectal (88.3%), bronchopulmonary (73.5%), and appendiceal (71.0%) carcinoids 2.

## 3. FUNCTIONING AND NON-FUNCTIONING TUMOURS

Neuroendocrine tumours may be benign or malignant, and functioning (that is, exhibiting excessive hormone production) or non-functioning 6. Carcinoid tumours are usually slow-growing, and patients with gastrointestinal carcinoids may be asymptomatic or may have vague gastrointestinal complaints that are often diagnosed as irritable bowel syndrome. Nonfunctioning tumours are usually found incidentally during surgery, and their neuroendocrine origin may be recognized only after histologic examination. Nonfunctioning pancreatic tumours can produce symptoms of a mass effect. The symptoms of intermittent intestinal entrapment seen with most midgut tumours are attributable to mesenteric fibrosis 11.

Functional tumours secrete bioactive mediators, notably serotonin, resulting in the manifestations of carcinoid syndrome, which is characterized by flushing, diarrhea, bronchoconstriction, and palpitations 12–15. Excess serotonin appears to be the major contributor to cardiac damage (endomyocardial fibrosis, tricuspid insufficiency, and pulmonary valvular disease), and carcinoid-related heart disease can have fatal consequences.

Numerous neuropeptides have also been found in excess in patients with carcinoid tumour. In fact, there is speculation that one or more combinations of these neuropeptides may be major contributors to the occurrence of carcinoid—and especially to flushing and other vasoactive manifestations.

The severity of carcinoid syndrome depends on tumour size and extent of metastases, especially the presence of liver metastases or others with direct access to the systemic (as opposed to the portal) circulation. Metastases are indicative of advanced disease.

## 4. BIOCHEMICAL MARKERS

Conventional tumour pathology criteria and dna cytometry have limited value in assessing the malignancy of a neuroendocrine tumour. Hence, the detection of substances that are more specific for carcinoid tumours can facilitate a more exact diagnosis. Two markers are primarily used to diagnose and follow carcinoid tumours: 5-hydroxy-indole-acetic acid (5-hiaa) and chromogranin A (CgA).

### 4.1 5-HIAA

Serotonin released by carcinoid tumours is metabolized by monoamine oxidases in the liver, lungs, and brain to 5-hiaa. When measured in a 24-hour urine sample, 5-hiaa level has a sensitivity of 73% and a specificity of 100% for diagnosing carcinoid 16. The normal range for urinary 5-hiaa is 3–15 mg/24 h, but the figure may vary depending on the laboratory. Up to 45 mg/24 h is considered normal by some laboratories. Levels of 5-hiaa have no clear correlation with symptoms, but they do fluctuate with symptomatology.

Additionally, 5-hiaa levels reflect the actions of somatostatin analogues, with a 50% reduction from pretreatment levels being indicative of a biochemical response. However, some patients with carcinoid tumour have symptoms of flushing with low or normal levels of 5-hiaa 17,18.

### 4.2 CgA

Chromogranin A is found in the wall of synaptic vesicles that store serotonin and glucagons. Levels of CgA tend to correlate with tumour bulk but not with symptoms. In general, CgA levels are elevated in 85%–100% of patients with carcinoid tumour, regardless of whether the tumour is functional or nonfunctional. The specificity of CgA has been found to be 98.4%, and the sensitivity, 62.9% 17–19.

In classical midgut neuroendocrine tumours, CgA levels are elevated to 100–1000 times normal 20,21. Measurements of CgA can be helpful, particularly if 5-hiaa is negative, but they are not as specific as measurements of 5-hiaa. Inflammatory conditions and renal insufficiency can cause elevations of CgA and, in rare cases, the cause cannot be identified. Type A gastritis and treatment with proton pump inhibitors can raise CgA levels. Change over time may be more useful than a single CgA value, because CgA levels are independent of symptoms. Practical problems, such as availability of the test, may hinder its use. Furthermore, several different methods for determining CgA are available, and the choice of method may affect the results. Chromogranin A appears to undergo a process of fragmentation, and the fragments detected by particular tests influence the resulting sensitivity.

### 4.3 Ki67

The Ki67 antigen is a nuclear protein expressed by proliferating cells; it is absent in resting cells. Expression can be tested in resected tissue specimens. Antibodies to Ki67 are a reliable marker of cell proliferation 22.

Assessment of Ki67 expression or antibody levels can be useful if chemotherapy is a consideration. High values (>2%) have some prognostic value for proliferation; patients with low values (<2%) are unlikely to benefit from chemotherapy 23. Expression or antibody levels of Ki67 should be tested in all patients.

### 4.4 Recommendations

- Levels of 5-hiaa should be measured at baseline and at 3- to 4-month intervals in the first year. If the patient is unstable or symptomatic, if evidence of disease progression is found, or if a change in therapy is being considered, measurement of the 5-hiaa level should be repeated.
- In the second year, the frequency of 5-hiaa measurements will depend on the patient’s status. If the patient is stable, measurements at 6-month intervals may be appropriate. If the patient has had a complete macroscopic resection, a measurement every year may be adequate.
- Where the test is available, CgA should be measured every 3 months in the first year. Elevated CgA in the absence of other altered parameters generally warrants further investigation.

### 4.5 Points for Further Discussion

- Centres and techniques for CgA.

## 5. DIAGNOSTIC IMAGING

Radiographic and nuclear imaging play important roles in the diagnosis and management of carcinoid tumours. Coupling a radioisotope to a molecule that specifically binds to receptors on tumour cells or that is selectively transported into tumour cells [for example, meta-iodobenzylguanidine (mibg)] can facilitate imaging and can also make it possible to deliver a radiation dose to the tumour without damaging non-tumour cells. After an injection of a radionuclide such as ^111^In-pentetreotide or ^123^I- or ^131^I-mibg, a gamma camera can visualize neuroendocrine tumours and their metastases in the body.

Scintigraphy using ^111^In-pentetreotide is one of the most important imaging investigations for identifying and staging carcinoid tumours of the gastrointestinal tract. Use of ^123^I-mibg can help to identify chromaffin cell tumours. Helical computed tomography (ct), magnetic resonance imaging (mri), and ultrasonography are used for determining the precise location of tumours and for monitoring response to treatment. Positron emission tomography (pet) scanning may be used for localizing a tumour when other imaging techniques have failed. Usually, pet is not useful because of the low metabolic rate of carcinoid tumours. However, in the future, if neuroendocrine-specific markers are used, pet may become the most sensitive technique.

### 5.1 ^111^In-Pentetreotide Scintigraphy

Somatostatin is a peptide that acts as an inhibitory factor by binding to somatostatin receptors. Because somatostatin has a short half-life (1–2 minutes), long-acting analogues such as octreotide and lanreotide have been synthesized. An ^111^In-labelled somatostatin analogue, pentetreotide, which shares the receptor-binding profile of octreotide, concentrates in neuroendocrine and some non-neuroendocrine tumours containing somatostatin receptor subtypes 2 and 5, making it suitable for the radio-imaging of tumours containing those somatostatin receptor subtypes. It is highly sensitive and specific for carcinoid tumours and has similar specificity for both functioning and non-functioning tumours.

OctreoScan (Mallinckrodt, St. Louis, MO, U.S.A.) can show early evidence of lesions not revealed by other procedures. The results are particularly helpful if surgery is being considered. Avidity on OctreoScan indicates a positive response to octreotide and is useful in planning treatment 24–27.

All patients should have an OctreoScan as a baseline, and patients who have undergone curative surgery should have an annual OctreoScan, unless clinical conditions indicate otherwise. For example, if a ct scan of the liver shows disease progression, an earlier OctreoScan may be required to see if the disease is progressing to other sites.

### 5.2 MIBG Scintigraphy

Meta-iodobenzylguanidine concentrates in carcinoid tumours, and occasionally a carcinoid tumour will take up mibg and not octreotide. Hence, ^123^I- or ^131^I-labelled mibg, used in place of octreotide, is increasingly recommended as a diagnostic test for carcinoid tumours. Imaging quality is superior with ^123^I-mibg, currently making it the radiopharmaceutical of choice 28. Scintigraphy using mibg is useful for imaging the extent of a tumour and in particular for distinguishing between primary and metastatic tumours. Only 80%–90% of carcinoid tumours express somatostatin receptors, and mibg can detect carcinoid tumours that are negative on OctreoScan. However, OctreoScan is considered to be the superior diagnostic test. Radiolabelled mibg testing is useful in identifying patients who can benefit from mibg therapy 27,29,30.

### 5.3 Conventional Imaging

Conventional imaging—such as triphasic ct, mri, and ultrasound—may also be helpful. A ct scan can measure disease that is not visible on an OctreoScan, and it may be sufficient to evaluate a tumour prior to radio-frequency ablation. Magnetic resonance imaging is not routinely employed in carcinoid tumour, but it can be helpful if ct or ultrasound results are conflicting or unhelpful.

Another new non-invasive technique for detection of carcinoid tumours in the future may be pet 27,31,32—if the appropriate tracers become available in Canada. Conventional pet with fluorodeoxy-glucose (fdg) detects only less well–differentiated neuroendocrine tumours and not the common well-differentiated neuroendocrine tumours.

### 5.4 Recommendations

- All patients should have an OctreoScan as a baseline.
- When no uptake is seen on OctreoScan, a scan with mibg should be considered if mibg is being considered as a part of therapy.
- The radiologist should be made aware of the objectives of the imaging and the possible treatment options and associated benefits.
- Patients who have undergone curative treatment should have an OctreoScan every year, unless clinical condition indicates otherwise.
- A scan using fdg-pet should be considered only for moderately- and poorly-differentiated neuroendocrine tumours and not for well-differentiated tumours.

### 5.5 Points for Further Discussion

- Any differences between ^123^I- or ^131^I-labelled mibg, and which one to use.
- Centres where mibg is available.

## 6. PRINCIPLES OF MANAGEMENT

The main goals of carcinoid tumour management are symptom control, biochemical control (that is, normalizing the 5-hiaa level), objective tumour control, and quality-of-life improvement. Different strategies are used to manage functioning and non-functioning carcinoid tumours. Somatostatin analogues for symptom control, frequent debulking, and better management of cardiac complications have improved survival rates.

Debulking (cytoreductive surgery) is a mainstay of therapy. Resection of the tumour can be curative. In widely metastatic disease, debulking and bypassing procedures can facilitate medical treatment. Debulking, laser treatment of metastases, radio-frequency ablation and embolization of liver metastases (either plain or combined with cytotoxic agents), liver resection, and, more recently, liver transplant (in selected patients) are possible options 3–6.

Somatostatin analogues such as octreotide and lanreotide have hormone-blocking properties and can facilitate the surgical procedure, provide symptom control, and reduce the risk of carcinoid crises and other severe events. They are first-line agents in the management of carcinoid syndrome. Alternatives in management include various combinations of isotope therapy, addition of interferon, and use of chemotherapy.

Somatostatin analogues control hypersecretion of neuropeptides in foregut and midgut carcinoid tumours that express somatostatin receptors, stabilizing the symptoms and possibly contributing to patient survival. Initially, immediate-release octreotide 150 μg is administered subcutaneously three times daily. Once tolerability and efficacy are established (during 3–7 days), 20–30 mg (and up to 60 mg) of the long-acting formulation (Sandostatin lar Depot: Novartis Pharmaceuticals Corporation, East Hanover, NJ, U.S.A.) is administered intramuscularly once per month. Immediate-release octreotide may be used to prevent breakthrough symptoms that may occur during lar therapy or with procedures such as chemoembolization or surgery.

### 6.1 Cytoreductive Surgery

Because of the slow growth and relatively favourable prognosis of carcinoid tumour, some clinicians may avoid surgery. However, the most common cause of death is advanced metastases, and long-lasting benefit can be obtained by disease ablation. Cyto-reductive surgery, which aims to control symptoms and improve survival by removing or destroying the tumour, is a mainstay in the management of carcinoid tumour 1,20,23,33. It may include tumour resection, radio-frequency ablation, and cryotherapy. For smaller tumours, local excision may be successful, but for larger tumours (>2 cm) more radical surgery may be required 34,35. In very select cases, liver transplantation has been performed.

Surgery is the only way to obtain a complete cure. Surgical resection of the primary tumour in midgut carcinoids and in cases with lymph node or liver involvement can improve survival. A delay in resection of a primary in the bowel, even if metastases have been identified, will make later attempts at resection more difficult. In the presence of metastases, surgery can improve hormone-related symptoms and quality of life, and can prolong survival in some patients. It reduces tumour bulk and prevents further local or systemic effects 23. Even if resection is not curative, it can be palliative. A meta-analysis of cytoreductive partial hepatectomy in patients with malignant carcinoid tumours showed a 5-year survival rate of 71% and complete resolution of carcinoid syndrome symptoms lasting 4–120 months in 86% of cases 36.

Some surgeons might hesitate to undertake such surgery. This hesitation may be related to difficulty in resecting the liver or to a lack of understanding of the value of cytoreduction in this tumour type. It is therefore worthwhile to identify a surgeon who is familiar with these procedures and their purpose.

Octreotide administration is crucial before an invasive procedure. In patients whose symptoms are well controlled with the long-acting form, a supplemental dose should be given 1–2 hours before the procedure. For emergency surgery, octreotide 500–1000 μg by intravenous bolus or 500 μg subcutaneously can be given 1–2 hours before the procedure, followed by an intravenous infusion 50–200 μg/h. At the time of surgery, a prophylactic cholecystectomy should be considered to mitigate the biliary toxicity of somatostatin analogue therapy 20,37.

#### 6.1.1 Recommendations

- Surgery should be considered, if technically feasible and clinically appropriate, to achieve cure or maximal debulking and palliation of symptoms.
- All patients should receive cytoreductive surgery at the earliest opportunity.
- In view of the potential benefits of surgery, reluctant surgeons may have to be persuaded to undertake surgery (consensus category 2B), or the patient may have to be referred to a surgical specialist who is experienced and knowledgeable about neuroendocrine tumour surgery.
- Short-acting octreotide should be given before invasive surgery.

### 6.2 Liver Metastases

The usual sites for metastases of carcinoid tumour are liver and bone. Central nervous system involvement is rare, except in the case of thymic carcinoids. For liver metastases, surgical intervention can be palliative. Surgical approaches include atypical liver resection, segmental resection, hemihepatectomy, and extended hemihepatectomy. The approach must be individualized for each patient. Surgical resections may be combined with other ablation techniques, particularly radio-frequency ablation. Intraoperative ultrasonography should be used to detect metastases and to determine their relationship to the hepatic vessels and bile ducts. For multiple liver metastases, options include debulking surgery, radio-frequency ablation, cryoablation, laser therapy, radiologic chemoembolization, medical therapy, or a combinations of these 38–40.

Liver transplantation may be an option for young patients from whom all extrahepatic tumours and metastases have been removed and in whom no recurrence of extrahepatic tumour is expected. However, in patients who have undergone liver transplantation, tumours have been found to recur within months or years 41.

#### 6.2.1 Recommendations

- Surgical treatment by surgeons with expertise and experience should be considered for liver metastases.
- The approach must be individualized for each patient.

### 6.3 Embolization of Liver Metastases

Because many patients have liver metastases at the time of diagnosis, treatment aimed at reducing tumour bulk in the liver can prolong survival. Embolization, either alone or in combination with intra-arterial chemotherapy (chemoembolization), can reduce clinical symptoms and liver metastases.

An intra-arterial injection of a cytotoxic drug [for example, 5-fluorouracil (5-fu), doxorubicin, or mitomycin C] is administered together with non-polar contrast. The drug concentrations achieved locally may be 20 times those achieved with systemic intravenous injection 3,23,42. Embolization of the supply artery prevents the contrast–chemotherapy from being ejected from the tumour vasculature, and it ultimately interferes with the blood supply to the tumour. A chemical response is noted in 70%–90% of patients, and significant tumour reduction in 30% –50% of patients, with symptomatic response lasting 15–30 months.

The embolization procedure is associated with a mortality rate that may be as high as 7% 3. Contra-indications to liver embolization are complete portal vein obstruction and hepatic insufficiency, involvement of more than 50% of liver volume, ascites, biliary tract “plumbing,” tumours larger than 7 cm, and non-permissive vascular supply. Major side effects include gallbladder necrosis, acute renal failure, pancreatitis, and liver abscess.

Details of the procedure, such as the timing of sequential chemoembolization and choice of cytotoxic drugs, are still not clear. More recently, the radio-contrast agent lipiodol has become available with ^131^I instead of cold iodine, permitting embolization with radioactive material. Bland embolization versus chemoembolization for hepatocellular carcinoma has been evaluated in three trials, with a significant advantage for chemoembolization being observed. No such trials are available for neuroendocrine carcinoma.

#### 6.3.1 Recommendations

- Where facilities and expertise exist to deliver the therapy, chemoembolization can be employed to reduce clinical symptoms and control liver metastases (consensus category 2B).

### 6.4 Medical Treatment

Systemic treatment for carcinoid tumours includes therapy with somatostatin analogues, interferon alfa, and cytotoxic agents. Other agents, such as loperamide or diphenoxylate for diarrhea and H_1_ or H_2_ blockers (or both) for histamine-secreting tumours may be administered as required.

#### 6.4.1 Somatostatin Analogues

Somatostatin analogues can relieve symptoms and reduce hormone levels. They are administered subcutaneously every 6–12 hours. Long-acting formulations require infrequent administration and have contributed to an improved quality of life for patients. Somatostatin analogues may be considered for asymptomatic patients with progressive disease, and they should be used to prevent or treat carcinoid crises before, during, and after procedures such as surgery and embolization.

Treatment usually involves the initial subcutaneous administration of immediate-release octreotide, with the long-acting formulation being given after tolerability and efficacy are established, usually over 3–7 days. Lifelong treatment is likely. Immediate-release octreotide may also be employed to prevent breakthrough symptoms occurring with procedures such as chemoembolization or surgery 1,3,20,23.

Tachyphylaxis and resistance to octreotide are known to occur. However, it is important to recognize whether an apparent lack of response is being caused by other factors. For example, patients who have had a right hemicolectomy or resection of the terminal ileum but who are not receiving cholestyr-amine may experience diarrhea. Their diarrhea may be attributed to carcinoid syndrome when it is actually due to irritation of the colon by bile acids that would normally have been absorbed in the terminal ileum. Likewise, diarrhea in patients who have had multiple bowel resections may be due to short bowel syndrome, which responds to dietary modification.

Occasionally, patients who are not tolerating octreotide may benefit from another somatostatin analogue, lanreotide. Conversely, patients failing lanreotide therapy may benefit from octreotide. Patients not responding to octreotide may also be candidates for other therapeutic measures, such as debulking, hepatic embolization, and radio-frequency ablation 20.

#### 6.4.2 Interferon Alfa

Interferon alfa inhibits protein and hormone synthesis in tumour cells, inhibits angiogenesis, and stimulates the immune system. It is a primary medical treatment for low-proliferating gastroenteropancreatic tumours, either alone or in combination with somatostatin analogues. It may be used as second-line therapy after cytotoxic treatment, alone or in combination with somatostatin analogues. The combination of interferon alfa with somatostatin analogues has additive and possible synergistic effects in treating classical carcinoids. Interferon alfa upregulates the expression of somatostatin receptors, and somatostatin analogues reduce the side effects of interferon alfa 3,43.

#### 6.4.3 Cytotoxic Treatment

Cytotoxic treatment is usually employed for tumours with a high proliferative capacity and large tumour burden (proliferation index by Ki67 antibody > 5%–10%). Highly proliferating tumours, such as malignant thymic and bronchial carcinoids, derive the maximum benefit; midgut tumours with low proliferation (Ki67 antibody < 2%) may not benefit from cytotoxic therapy. Single-agent cytotoxic treatment produces limited benefit, with response rates of less than 30%.

The combination of streptozotocin, 5-fu, and doxorubicin has shown a response rate of more than 50% in malignant pancreatic tumours, but malignant pulmonary, colorectal, and classical midgut carcinoids respond poorly to this combination. Poorly differentiated tumours of the foregut (pulmonary, thymic) and small-cell colorectal tumours have a high proliferation capacity (Ki67 antibody > 15%), and may respond to cisplatin/paraplatin plus etoposide 3,23. Poorly differentiated neuroendocrine tumours have shown a 67% response rate to etoposide plus cisplatin, but the prognosis is poor, with a 2-year survival of less than 20% 44–46. Other regimens that have been used include epirubicin, cisplatin, and fluorouracil; and imatinib, a new tyrosine-kinase inhibitor; but results with these regimens are inconclusive.

It needs to be recognized that carcinoid tumours in particular are often highly desmoplastic, and therefore standard endpoints such as tumour response by mensuration are not valid evaluations of the benefits of anti-neoplastic therapy. Imaging will visually reflect the consequent tumour-induced fibrosis and not the tumour itself. A more valid approach is therefore to assess tumour markers, ectopic hormone secretion, tumour-related symptoms, and organ function of metastatically-involved viscera.

#### 6.4.4 Recommendations

- Somatostatin analogues
  - are the primary treatment for carcinoid tumours associated with production of peptides. For patients with insulinoma and gastrinoma, they may be second- or third-line agents.
  - may be considered for asymptomatic patients with progressive disease.
  - should be used to prevent or treat carcinoid crises before, during, and after procedures such as surgery and embolization.
- Standard dosages of somatostatin analogues are
  - octreotide: short-acting form, 100–500 μg three times daily; long-acting form, 10–30 mg (up to 60 mg) every 4 weeks; and
  - lanreotide: slow release form, 60–120 mg every 4 weeks.
  - Interferon alfa (alone or in combination with somatostatin analogues)
  - is a primary medical treatment for low-proliferating gastroenteropancreatic tumours.
  - may be a second-line therapy after cytotoxic treatment.
  - should be titrated individually in each patient. Aim for reduction of leukocyte count to about 3.0×10^9^/L. The usual dosage of regular interferon alfa is 3–5 million units subcutaneously, 3 –5 times weekly. The dose of pegylated interferon alfa is not yet established, but may be 75–150 μg subcutaneously per week.
- Cytotoxic treatment
  - may be a first-line treatment for malignant neuroendocrine tumours in the pancreas and for gastric carcinoids if Ki67 antibody level is greater than 10%.
  - may be a second-line treatment if other means of treatment fail, and after tumour-targeted radioactive treatment.
  - agents are used at these dosages: 5-day induction with streptozotocin 1 g daily intravenously for 5 days, plus 5-fu 400 mg/m^2^ on days 1 3. Thereafter, streptozotocin 2 g and 5-fu 400 mg/m^2^ intravenously during 1 day every 3 weeks until toxicity develops. If doxorubicin is combined with streptozotocin, 40 mg/m^2^ is given on day 3. One or two years after stabilization, the interval between courses may be increased to 4–6 weeks (consensus category 2B).

## 7. RADIOTHERAPY: EXTERNAL BEAM AND RADIOISOTOPE THERAPY

External radiation therapy has limited value in carcinoid tumours; it is recommended only for bone and brain metastases. Tumour-targeted treatment with radioactive octreotide derivatives—that is, ^111^In–d-Phe(1)-Tyr(3)-octreotide (^111^In-dota-octreotide) or ^90^Y-dota-octreotide and ^177^Lu-dota-octreotate, is associated with tumour shrinkage in 20%–25% of tumours and with biochemical and clinical responses in 40%–50% of patients. In classical midgut carcinoids, ^131^I-mibg can produce biochemical responses in 30%–40% of patients and tumour responses in about 20%. However, the exact role of tumour-targeted radioactive treatment is not yet defined 3. It is nonetheless an accepted alternative in the therapy of these neoplasms. Some recent evidence suggests that radioisotope in combination with concurrent chemotherapy may be more efficacious.

## 8. MONITORING AND FOLLOW-UP

In the first year after diagnosis, patients should be monitored every 3–4 months to establish the “pace” of the disease. At each visit, conventional imaging (triphasic ct, mri, or ultrasonography) should be done, and urinary 5-hiaa and serum CgA (and possibly mibg) should be monitored. If a baseline OctreoScan is positive, an OctreoScan once yearly is recommended—or earlier if evidence of disease progression is seen. In the symptomatic patient, the follow-up schedule will depend on the aggressiveness of the disease.

In the second and subsequent years, visits every 4–6 months are generally appropriate, with an annual OctreoScan. If surgery, radiation, or a change in treatment modality is considered, an OctreoScan or mibg should be conducted. In the asymptomatic patient, follow-up involves watchful waiting to ensure that no transformation to more aggressive disease occurs.

Patients who have had a complete macroscopic resection should have a follow-up every year, with annual evaluation of 5-hiaa, CgA, and mibg if available. If any result is positive, a ct scan and OctreoScan should be considered.

The use of traditional radiologic methods to assess the response of a neuroendocrine tumour yields poor results, particularly within the liver. The World Health Organization and the Response Evaluation Criteria in Solid Tumours criteria are based largely on assessment of serial measurements of tumour size before and after treatment, and are used to define tumour regression.

### 8.1 Recommendations

- In the first year after diagnosis,
  - patients should be monitored every 3–4 months to establish the “pace” of the disease.
  - conventional imaging (triphasic ct, mri, or ultrasound), urinary 5-hiaa monitoring, and serum CgA (and possibly mibg) should be done at each visit.
- In subsequent years, visits every 4–6 months are appropriate.
- An annual OctreoScan is recommended unless more frequent evaluation is indicated. An OctreoScan may be performed earlier if evidence of disease progression is seen.
- In the symptomatic patient,
  - the follow-up schedule will depend on the aggressiveness of the disease.
  - in the second and subsequent years, follow-up visits every 4–6 months are appropriate, with an annual OctreoScan.
  - if surgery, radiation, or a change in treatment modality is considered, an OctreoScan or mibg should be conducted.
- In the asymptomatic patient,
  - follow-up involves watchful waiting to ensure no transformation to more aggressive disease occurs.
  - after complete macroscopic resection, follow-up should be annual, with an annual evaluation of 5-hiaa, CgA, and mibg if available.
  - if any results are positive, a ct and Octreo-Scan should be considered.

### 8.2 Points for Further Discussion

- Should 5-hiaa be controlled with octreotide even in asymptomatic patients?
- Should the dose of octreotide be increased to bring 5-hiaa to within normal values?

## 9. CARCINOID HEART DISEASE

Carcinoid syndrome is associated with the release of serotonin and other vasoactive substances by the carcinoid tumour. Exposure of the heart to high levels of these substances can result in endocardial damage, manifesting as right-sided endomyocardial fibrosis, valvular insufficiency, and heart failure. The most common lesion is tricuspid regurgitation, followed by tricuspid stenosis, pulmonary regurgitation, and pulmonary stenosis. Right ventricular failure is a major cause of morbidity and mortality in carcinoid heart disease 47,48. Cardiac involvement is detected by echocardiography in more than 50% of patients with carcinoid syndrome 23,49.

Carcinoid heart disease occurs in asymptomatic patients and in those with small increases in 5-hiaa, but it is more commonly associated with chronic elevation of 5-hiaa. The role of serotonin in the development of carcinoid-related heart disease is controversial, but the indirect evidence appears compelling. Only carcinoid patients with elevated 5-hiaa levels develop this complication. Serotonin receptors have been identified in the heart. Experimentally, stimulation of these receptors causes cardiac cell proliferation. Two drugs, fen-phen and methysergide, can cause identical cardiac pathology. Both have chemical structures analogous to serotonin, and both can be shown to bind to heart serotonin receptors. Recent evidence suggests that both serotonin and atrial natriuretic factor may be required for the lesions to occur. Brain natriuretic peptide may be a useful prognostic marker 50.

Carcinoid heart disease is likely to develop in patients with long-standing elevations of 5-hiaa (>75 mg/24 h) and is almost never seen in patients with 5-hiaa values below 50 mg/24 h 51–54. Patients with 5-hiaa levels greater than 50 mg/24 h should be considered for octreotide therapy (consensus category 2B). Tachyphylaxis with octreotide is more common in patients with a larger tumour burden.

The symptoms of carcinoid heart disease may be so subtle as to be attributed to non-cardiac causes. Hence, it is advisable that patients with carcinoid syndrome have an echocardiogram at diagnosis. Patients who are predisposed to carcinoid heart disease should have an annual echocardiogram and follow-up with a cardiologist.

The use of somatostatin analogues, titrated to manage symptoms or to normalize 5-hiaa levels, can help to prevent or minimize carcinoid-related heart disease, but such use is controversial. Carcinoid heart disease may continue to progress even if 5-hiaa is carefully controlled 48.

Once carcinoid heart disease is diagnosed, treatment should be initiated early. Instituting treatment after the patient has developed pump failure is associated with higher mortality. General measures for heart failure include restricting salt and water intake and monitoring fluid balance and weight. Right-sided heart failure is managed with loop diuretics and digoxin. A thiazide diuretic may be judiciously co-administered if additional diuresis is required 55.

Cytoreductive surgery to reduce the number of serotonin receptors can be beneficial 33. Unfortunately, it may be difficult to find a cardiologist who is interested in treating a patient who has cancer. Some patients with carcinoid heart disease may benefit from cardiac valve replacement 56, but cardiac surgeons may be reluctant to treat these patients. In such circumstances, the best approach involves close monitoring of predisposed patients and aggressive treatment to prevent heart failure.

### 9.1 Recommendations

- Patients with carcinoid syndrome should have an echocardiogram at diagnosis.
- All patients should have an annual echocardiogram and follow-up with a cardiologist, particularly those with any cardiac changes or with elevated 5-hiaa.
- Patients with 5-hiaa levels greater than 50 mg/24 h should be considered for octreotide therapy (consensus category 2B). Because long-term, low-level exposure to serotonin may cause cardiac complications, normalization of 5-hiaa 24-hour urine excretion is an appropriate goal, if it can be achieved.
- For carcinoid-related heart disease, the morbidity and mortality rate is so high that early intervention with octreotide should be considered, given that toxicity is low.

### 9.2 Point for Discussion

- Patients with 5-hiaa levels greater than 50 mg/ 24 h should be considered for octreotide therapy.

## 10. NEW AGENTS

New agents such as imatinib may be of benefit in selected patients. Anti-angiogenic substances such as Endostatin (Entremed, Rockville, MD, U.S.A.), angiostatin, and the new compound 2004-01-13IZD6126 may have future roles. A new somatosta-tin analogue, som230, which binds to somatostatin receptors 1, 2, 3, and 5, could be interesting.

## Figures and Tables

**Table I:** Categories of consensus^a^

Category 1	Uniform consensus based on high-level evidence that the recommendation is appropriate.
Category 2A	Uniform consensus based on lower level evidence, including clinical experience, that the recommendation is appropriate.
Category 2B	Non-uniform consensus, but no major disagreement, based on lower-level evidence, including clinical experience, that the recommendation is appropriate.
Category 3	Major disagreement that the recommendation is appropriate.

a All recommendations in this statement are category 2A unless otherwise indicated.

## References

[1] Schnirer II, Yao JC, Ajani JA (2003). Carcinoid—a comprehensive review. Acta Oncol.

[2] Modlin IM, Lye KDA, Kidd M (2003). A 5-decade analysis of 13,715 carcinoid tumours. Cancer.

[3] Öberg K, Astrup L, Eriksson B, for the Nordic NE Tumour Group (2004). Guidelines for the management of gastroenteropancreatic neuroendocrine tumours (including bronchopulmonary and thymic neoplasms). Part i—general overview. Acta Oncol.

[4] Öberg K, Astrup L, Eriksson B, for the Nordic NE Tumour Group (2004). Guidelines for the management of gastroenteropancreatic neuroendocrine tumours (including bronchopulmonary and thymic neoplasms). Part ii—specific NE tumour types. Acta Oncol.

[5] Plöckinger U, Rindi G, Arnold R (2004). Guidelines for the diagnosis and treatment of neuroendocrine gastrointestinal tumours. A consensus statement on behalf of the European Neuroendocrine Tumour Society (enets). Neuroendocrinology.

[6] National Comprehensive Cancer Network (nccn). Clinical Practice Guidelines in Oncology: Neuroendocrine Tumours, Version 2.2005 [Web site]. Jenkintown, PA: nccn; 2005. [Available at: www.nccn.org/professionals/physician_gls/PDF/neuroendocrine.pdf; cited August 30, 2005]

[7] Ramage JK, Davies AH, Ardill J, for the UKNETwork for Neuroendocrine Tumours (2005). Guidelines for the management of gastroenteropancreatic neuroendocrine (including carcinoid) tumours. Gut.

[8] Crocetti E, Paci E (2003). Malignant carcinoids in the U.S.A., seer 1992–1999. An epidemiological study with 6830 cases. Eur J Cancer Prev.

[9] Modlin IM, Lye KD, Kidd M (2004). A 50-year analysis of 562 gastric carcinoids: small tumour or larger problem?. Am J Gastroenterol.

[10] Zar N, Garmo H, Holmberg L, Rastad J, Hellman P (2004). Long-term survival of patients with small intestinal carcinoid tumours. World J Surg.

[11] Plöckinger U, Wiedenmann B (2004). Diagnosis of non-functioning neuroendocrine gastroenteropancreatic tumours. Neuroendocrinology.

[12] Kinova S, Duris I (2001). Carcinoid tumour. Bratisl Lek Listy.

[13] Bell HK, Poston GJ, Vora J, Wilson NJ (2005). Cutaneous manifestations of the malignant carcinoid syndrome. Br J Dermatol.

[14] van der Horst–Schrivers AN, Wymenga AN, Links TP, Willemse PH, Kema IP, de Vries EG (2004). Complications of mid-gut carcinoid tumours and carcinoid syndrome. Neuroendocrinology.

[15] Lips CJ, Lentjes EG, Hoppener JW (2003). The spectrum of carcinoid tumours and carcinoid syndromes. Ann Clin Biochem.

[16] Feldman JM, O’Dorisio TM (1986). Role of neuropeptides and serotonin in the diagnosis of carcinoid tumours. Am J Med.

[17] Eriksson B, Öberg K, Stridsberg M (2000). Tumour markers in neuroendocrine tumours. Digestion.

[18] Öberg K, Janson ET, Eriksson B (1999). Tumour markers in neuroendocrine tumours. Ital J Gastroenterol Hepatol.

[19] Nehar D, Lombard–Bohas C, Olivieri S (2004). Interest of chromogranin A for diagnosis and follow-up of endocrine tumours. Clin Endocrinol.

[20] Öberg K, Kvols L, Caplin M (2004). Consensus report on the use of somatostatin analogs for the management of neuroendocrine tumours of the gastroenteropancreatic system. Ann Oncol.

[21] Jensen TB, Hilsted L, Rehfeld JF (1999). Library of sequence-specific radioimmunoassays for human chromogranin A. Clin Chem.

[22] Sokmensuer C, Gedikoglu G, Uzunalimoglu B (2001). Importance of proliferation markers in gastrointestinal carcinoid tumours: a clinicopathologic study. Hepatogastroenterology.

[23] Öberg K (2003). Diagnosis and treatment of carcinoid tumours. Expert Rev Anticancer Ther.

[24] Frilling A, Malago M, Martin H, Broelsch CE (1998). Use of somatostatin receptor scintigraphy to image extrahepatic metastases of neuroendocrine tumours. Surgery.

[25] Kisker O, Bartsch D, Weinel RJ (1997). The value of somatostatin-receptor scintigraphy in newly diagnosed endocrine gastroenteropancreatic tumours. J Am Coll Surg.

[26] Kwekkeboom DJ, Krenning EP (1996). Somatostatin receptor scintigraphy in patients with carcinoid tumours. World J Surg.

[27] Kaltsas G, Rockall A, Papadogias D, Reznek R, Grossman AB (2004). Recent advances in radiological and radionuclide imaging and therapy of neuroendocrine tumours. Eur J Endocrinol.

[28] Kaltas GA, Besser GM, Grossman AB (2004). The diagnosis and medical management of advanced neuroendocrine tumours. Endocr Rev.

[29] Le Rest C, Bomanji JB, Costa DC, Townsend CE, Visvikis D, Ell PJ (2001). Functional imaging of malignant paragangliomas and carcinoid tumours. Eur J Nucl Med.

[30] Kaltsas G, Korbonits M, Heintz E (2001). Comparison of somatostatin analog and meta-iodobenzylguanidine radionuclides in the diagnosis and localization of advanced neuroendocrine tumours. J Clin Endocrinol Metab.

[31] Gibril F, Jensen RT (1997). Comparative analysis of diagnostic techniques for localization of gastrointestinal neuroendocrine tumours. Yale J Biol Med.

[32] Öberg K, Eriksson B (2005). Nuclear medicine in the detection, staging and treatment of gastrointestinal carcinoid tumours. Best Pract Res Clin Endocrinol Metab.

[33] Woodside KJ, Townsend CM, Mark Evers B (2004). Current management of gastrointestinal carcinoid tumours. J Gastrointest Surg.

[34] Loftus JP, van Heerden JA (1995). Surgical management of gastrointestinal carcinoid tumours. Adv Surg.

[35] Stinner B, Kisker O, Zielke A (1996). Surgical management for carcinoid tumours of small bowel, appendix, colon, and rectum. World J Surg.

[36] Que FG, Sarmiento JM, Nagorney DM (2002). Hepatic surgery for metastatic gastrointestinal neuroendocrine tumours. Cancer Control.

[37] Trendle MC, Moertel CG, Kvols LK (1997). Incidence and morbidity of cholelithiasis in patients receiving chronic octreotide for metastatic carcinoid and malignant islet cell tumours. Cancer.

[38] Sutcliffe R, Maguire D, Ramage J (2004). Management of neuroendocrine liver metastases. Am J Surg.

[39] Sarmiento JM, Heywood G, Rubin J (2003). Surgical treatment of neuroendocrine metastases to the liver: a plea for resection to increase survival. J Am Coll Surg.

[40] Sarmiento JM, Que FG (2003). Hepatic surgery for metastases from neuroendocrine tumours. Surg Oncol Clin N Am.

[41] Florman S, Toure B, Kim L (2004). Liver transplantation for neuroendocrine tumours. J Gastrointest Surg.

[42] Roche A, Girish BV, de Baere T (2003). Trans-catheter arterial chemoembolization as first-line treatment for hepatic metastases from endocrine tumours. Eur Radiol.

[43] Öberg K (2000). Interferon in the management of neuroendocrine gep-tumours: a review. Digestion.

[44] Moertel CG, Kvols LK, O’Connell MJ, Rubin J (1991). Treatment of neuroendocrine carcinomas with combined etoposide and cisplatin. Evidence of major therapeutic activity in the ana-plastic variants of these neoplasms. Cancer.

[45] Fjallskog ML, Granberg DP, Welin SL (2001). Treatment with cisplatin and etoposide in patients with neuroendocrine tumours. Cancer.

[46] Mitry E, Baudin E, Ducreux M (1999). Treatment of poorly differentiated neuroendocrine tumours with etoposide and cisplatin. Br J Cancer.

[47] Quaedvlieg PF, Lamers CB, Taal BG (2002). Carcinoid heart disease: an update. Scand J Gastroenterol.

[48] Moller JE, Connolly HM, Rubin J, Seward JB, Modesto K, Pellikka PA (2003). Factors associated with progression of carcinoid heart disease. N Engl J Med.

[49] Di Luzio S, Rigolin VH (2000). Carcinoid heart disease. Curr Treat Options Cardiovasc Med.

[50] Zuetenhorst JM, Korse CM, Bonfrer JM, Bakker RH, Taal BG (2004). Role of natriuretic peptides in the diagnosis and treatment of patients with carcinoid heart disease. Br J Cancer.

[51] Pellikka PA, Tajik AJ, Khandheria BK (1993). Carcinoid heart disease: clinical and echocardiographic spectrum in 74 patients. Circulation.

[52] Lundin L, Norheim I, Landelius J, Öberg K, Theodorsson–Norheim E (1988). Carcinoid heart disease: relationship of circulating vasoactive substances to ultrasound-detectable cardiac abnormalities. Circulation.

[53] Denney WD, Kemp WE, Anthony LB, Oates JA, Byrd BF (1998). Echocardiographic and biochemical evaluation of the development and progression of carcinoid heart disease. J Am Coll Cardiol.

[54] Robiolio PA, Rigolin VH, Wilson JS (1995). Carcinoid heart disease: correlation of high serotonin levels with valvular abnormalities detected by cardiac catheterization and echocardiography. Circulation.

[55] Fox DJ, Khattar RS (2004). Carcinoid heart disease: presentation, diagnosis, and management. Heart.

[56] Wilhelmi M, Fritz MK, Fischer S, Haverich A, Harringer W (2002). Triple valve replacement in a patient with severe carcinoid heart disease. Cardiovasc Surg.

